# Gel‐immersion Endoscopic Submucosal Dissection for Superficial Colorectal Neoplasms: A Retrospective Study Comparing Conventional Endoscopic Submucosal Dissection

**DOI:** 10.1002/deo2.70221

**Published:** 2025-10-10

**Authors:** Kenji Yamauchi, Tomoki Inaba, Takeshi Morimoto, Hugh Shunsuke Colvin, Akira Nakanishi, Shigenao Ishikawa, Masaki Wato

**Affiliations:** ^1^ Department of Gastroenterology Kagawa Prefectural Central Hospital Kagawa Japan; ^2^ Department of Clinical Epidemiology Hyogo College of Medicine Hyogo Japan

**Keywords:** colon neoplasms, endoscopic mucosal resection, endoscopic surgery, gravitation, immersion

## Abstract

**Objectives:**

Gel‐immersion endoscopy offers benefits such as buoyancy, traction, and a clear visual field without gas insufflation. While some case reports have described colorectal gel‐immersion endoscopic submucosal dissection (Gi‐ESD), there have been no consecutive case series. This study aimed to clarify the usefulness of Gi‐ESD.

**Methods:**

This single‐center retrospective cohort study included consecutive patients with colorectal neoplasms who underwent ESD. Gi‐ESD was defined as mucosal incision and submucosal dissection performed under clear gel. The primary outcomes were en bloc and histologic R0 resection rates, whereas the secondary outcomes were procedure time, dissection speed, and adverse events.

**Results:**

Among 260 ESD cases, 29 and 231 were in the Gi‐ESD and conventional ESD groups, respectively. Gel was used for submerged or poorly accessible lesions. The Gi‐ESD group had a significantly larger tumor diameter (25 mm vs. 18 mm, *p* = 0.001), a higher rate of lesions in the cecum or ascending colon (55.2% vs. 31.2%, *p* = 0.01), and more lesions with ESD difficulty factors (24.1% vs. 9.5%, *p* < 0.05). There were no significant differences in the en bloc resection (100% vs. 99.1%), histologic R0 resection (96.6% vs. 88.7%), or adverse events. In the propensity score‐matched cohort, the histologic R0 resection rate was significantly higher in the Gi‐ESD group (100% vs. 82.6%, *p* = 0.045). Procedure time was significantly longer in the Gi‐ESD group (45 vs. 29.5 min, *p* = 0.006), with no significant difference in dissection speed (14.9 vs. 19.2 mm^2^/min, *p* = 0.19).

**Conclusion:**

Gi‐ESD may be an alternative approach for treating submerged gravity‐side or poorly approached colorectal lesions.

## 1 Introduction

Endoscopic submucosal dissection (ESD) has become an established treatment for superficial colorectal neoplasms. Nevertheless, several challenges regarding difficult lesions remain, including lesions located on the gravity side that become submerged in fluids, lesions located in the flexures that are difficult to approach, and vertically oriented lesions that are difficult to access during the submucosal dissection. When performing ESD for lesions on the gravity side, gravity hinders submucosal elevation, making submucosal dissection difficult [1]. Furthermore, blood retention can obstruct the visual field, rendering hemostasis and dissection difficult. Lesions located in the flexures or with a vertical orientation require reduced gas insufflation and endoluminal pressure to allow the ESD knife to be in parallel to the muscularis propria during dissection, which often makes it difficult to maintain a favorable visual field.

VISCOCLEAR is a clear, viscous gel solution developed to secure the visual field during bleeding during endoscopic procedures [2]. The advantages of treatment under the gel called “gel‐immersion endoscopy” [3] include not only the easy identification of the bleeding point [4] but also less obstruction of the visual field by intestinal fluids and residues [5], buoyancy traction by floating lesions [6], good combination with the tapered tip hood due to its magnifying effect [6], less pain during the procedure [7], and ease in handling due to low pressure in the intestinal tract [8]. These effects are favorable for colorectal ESD, and there have been case reports of colorectal gel‐immersion ESD (Gi‐ESD) [4, 6]; however, there have been no consecutive case series studies. Therefore, we conducted a retrospective cohort study to assess the effectiveness of gel use in colorectal ESD.

## 2 Methods

### 2.1 Study Design and Patient Population

This was a retrospective cohort study of consecutive patients with colorectal neoplasms who underwent ESD between July 2022 and May 2024 at the Kagawa Prefectural Central Hospital, a tertiary care center.

The inclusion criterion was all colorectal ESD procedures performed at our hospital. The exclusion criteria were as follows: (i) non‐neoplastic lesions, (ii) carcinoma invading the muscularis propria, (iii) underwater ESD, (iv) use of traction devices, and (v)significant loss of clinical data. All lesions met the criteria recommended by the Japanese Colorectal ESD/EMR guidelines [9].

All lesions were examined for their degree of submersion in water prior to the procedure. The decision to use gel was made by the trainer. All surgeries were recorded, and the timing of using the gel and assistive devices (snares or knives other than the tip type) was investigated.

A lesion was deemed to be a “difficult case” when at least one of the following conditions was present: (i) the lesion involved the appendiceal orifice, ileocecal valve [10], diverticulum [11], dentate line [12, 13], post‐endoscopic resection scar [14], or tattooing [15]; (ii) the circumferential range was more than two‐thirds [13]; and (iii) the muscle‐retracting sign was present [16].

Intraoperative and postoperative perforations were defined as muscle propria damage with visible extraintestinal adipose tissue during ESD and extraintestinal air, respectively, as confirmed by computed tomography. Postoperative bleeding was defined as bloody stool with a >2 mg/dL reduction in the hemoglobin level. Treatment outcomes were compared between Gi‐ESD and conventional ESD (c‐ESD).

### 2.2 Gel‐immersion ESD and Conventional ESD

In this study, gel‐immersion ESD (Gi‐ESD) was defined as mucosal incision and submucosal dissection performed partially or totally under clear gel (VISCOCLEAR; Otsuka Pharmaceutical Factory, Tokushima, Japan). Additionally, c‐ESD was defined as all incisions and dissections performed without gel. All ESD procedures were performed under CO_2_ insufflation.

For both Gi‐ESD and c‐ESD, an endoscope (GIF‐H290T or PCF‐H290I, Olympus, Tokyo, Japan) with a water‐jetting function was used with a tapered transparent hood (ST hood short type, Fujifilm, Tokyo, Japan) attached to the tip of the endoscope. Hyaluronic acid (MucoUp; Boston Scientific, Tokyo, Japan) or an alginate solution (Riftal K; Kaigen Pharma Co., Ltd., Osaka, Japan) mixed with indigo carmine or glycerin was used for submucosal injection. VIO3 (Erbe Elektromedizin, Tübingen, Germany) was used as the electrosurgical generator. The Endocut I mode was used for mucosal incision, and the forced or spray coagulation mode was used for submucosal dissection. 1.5 mm Tip‐type knives (dual knife, Olympus, or Tech knife, Micro‐Tech Co., Ltd., Nanjing, China) were used for mucosal incision and submucosal dissection. Both Gi‐ESD and c‐ESD were performed using the pocket creation method [17], which first creates a large mucosal pocket under the tumor with a minimal mucosal incision.

**FIGURE 1:**
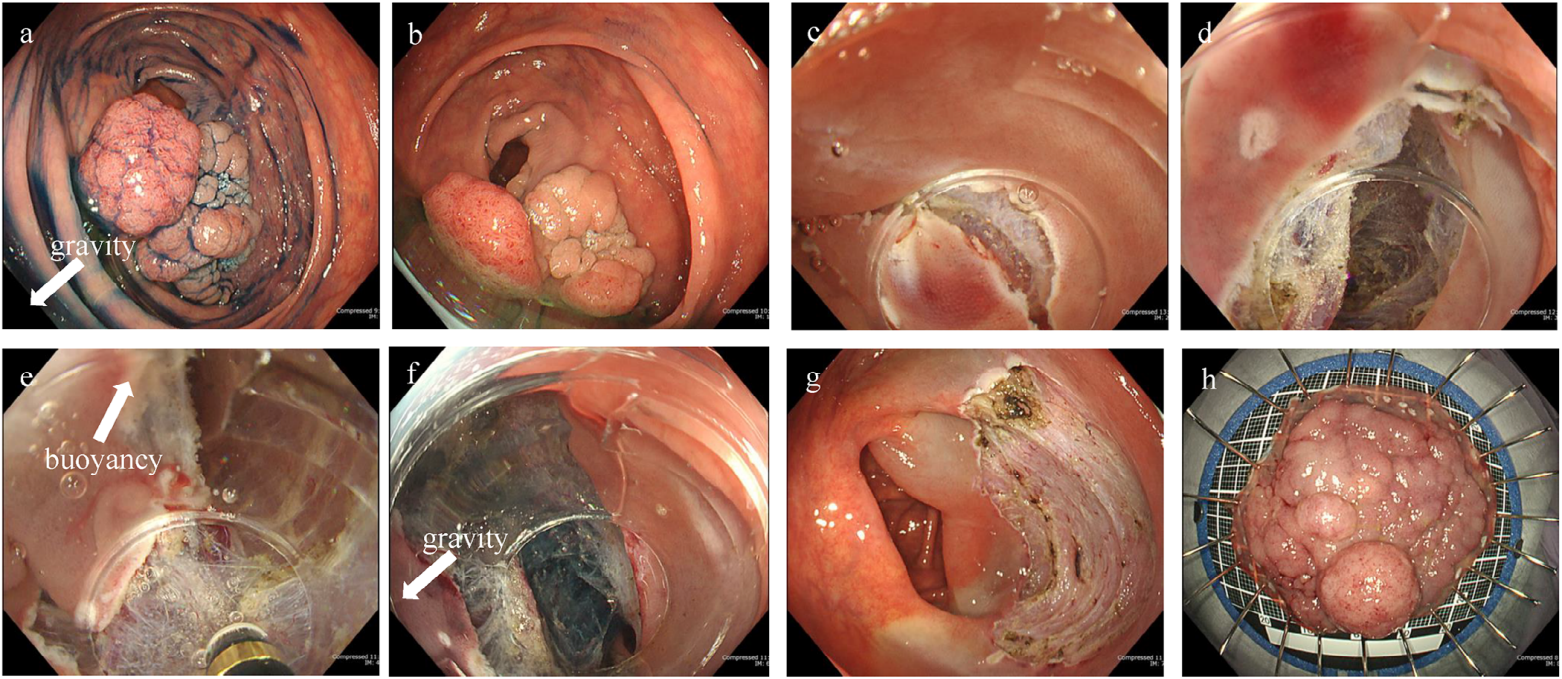
(a) Laterally spreading granular tumor (LST‐G) with a diameter of 50 mm was located on the ascending colon. (b) The downward side of the lesion was easily submerged. (c) Mucosal incision under the gel. (d) Creation of a submucosal pocket under the gel. (e) Opening the gravity side of the pocket using buoyancy traction. (f) Opening the remaining upward side under CO_2_ insufflation using gravity traction. (g) Mucosal defect without perforation. (h) En bloc R0 resection.

Additional gel was injected through the endoscopic large accessory channel with a BioShield irrigator (US Endoscopy (STERIS), Ohio, United States) when the gel viscosity decreased with a water jet or when intestinal fluid or bleeding obstructed the visual field (Figure 1 and Video S1). Considering the additional cost of the gel, gel use was limited to one pack (200 g) per case, with a maximum of two packs being used in exceptional cases.

Before starting ESD, the patient's posture was determined based on the direction of gravity, the approach angle of the scope, and maneuverability, following which sedation was administered. Throughout the procedure, the patient's posture was maintained.

All procedures were performed by one board‐certified trainer from the Japan Gastroenterological Endoscopy Society and one trainee. Concerning the surgeons, “only expert” was defined as the case where all the procedures were performed by the trainer, and the case where some or all of the procedures were performed by the trainer was defined as “with trainer.” Two trainers and five trainees participated in the study.

### 2.3 Data Collection and Variables

The baseline characteristics of the eligible patients were collected from medical records, including age, sex, endoscopic findings such as lesion location, growth type, submucosal fibrosis, and factors associated with technical difficulty in ESD, and pathological findings such as histopathology, tumor diameter, and major and minor resected specimen diameters. Submucosal fibrosis was classified endoscopically as none (F0), moderate (F1), or severe (F2) [18] during procedures.

### 2.4 Outcomes

The primary outcomes were en bloc and histologic R0 resection rates. En bloc resection was defined as the resection of the tumor‐containing specimen in one lump. Histologic R0 resection was defined as no tumor exposure at the lateral and deep resection margins. Resection was classified as R1 if residual lesions were present, and as RX with an indeterminate resection margin.

The secondary outcomes were procedure time, dissection speed, intraoperative or postoperative perforation, postoperative hemorrhage, and gel‐related adverse events. Procedure time was defined as the time from the initial mucosal incision to lesion removal. Dissection speed was calculated as follows: (major diameter of the resected specimen (mm)/2) × (minor diameter of the resected specimen (mm)/2) × 3.14/procedure time.

### 2.5 Statistical Analysis

Categorical variables were presented as numbers with percentages and compared using the chi‐square test or Fisher's exact test. Continuous variables were expressed as means with standard deviations or medians with interquartile ranges. Based on their distributions, continuous variables were compared using Student's t‐test or the Mann–Whitney U test. *p*‐Values were two‐sided, with statistical significance set at *p* < 0.05. For robustness, propensity score matching and subgroup analyses were performed. In propensity score matching, propensity scores were calculated based on tumor size, tumor location, circumferential location, fibrosis, ESD difficulty factors, and operator. One‐to‐two matching was performed using a caliper of 0.02 based on the propensity score. All statistical analyses were performed using EZR version 1.68 (Saitama Medical Center, Jichi Medical University, Saitama, Japan), a graphical user interface for R (The R Foundation for Statistical Computing, Vienna, Austria).

## 3 Results

### 3.1 Baseline Characteristics

**FIGURE 2:**
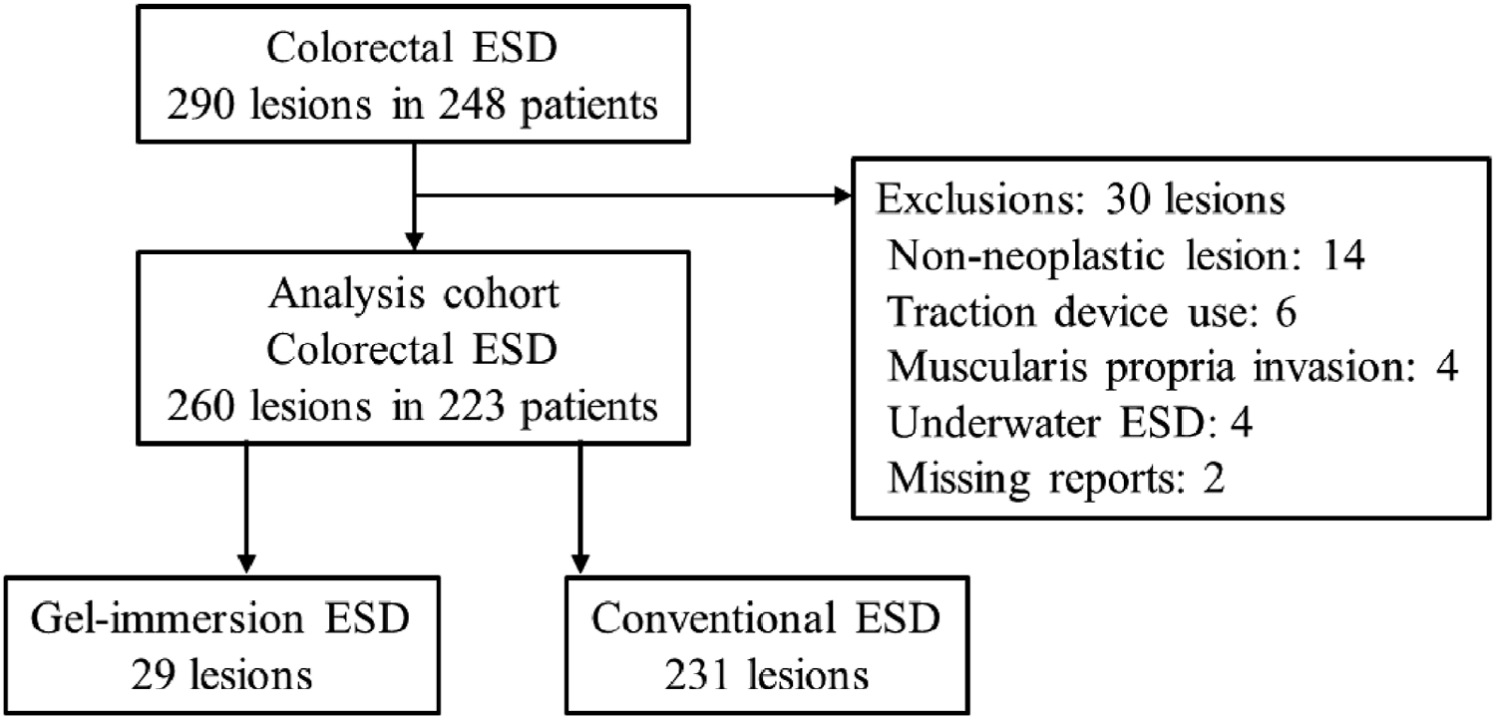
Study flowchart. ESD, endoscopic submucosal dissection.

Out of 290 colorectal ESD cases, 260 (223 patients) were included (reasons for exclusion: non‐neoplastic lesions, 14 lesions; traction device use, six; muscularis propria invasion, four; underwater ESD, four; missing reports, two). Among the 260 ESD cases, 29 and 231 were included in the Gi‐ESD and c‐ESD groups, respectively (Figure 2). The median patient age was 71 years, with male patients accounting for 57.8%. The Gi‐ESD group had a significantly larger tumor diameter (25 vs. 18 mm, *p* = 0.001), a higher rate of lesions located in the cecum or ascending colon, and a higher rate of lesions with factors associated with difficult ESD. The c‐ESD group had significantly more lesions located on the opposite side of gravity (23.4% vs. 0%, *p* = 0.002); that is, all or part of the lesions in the Gi‐ESD group were on the gravity side. The groups did not significantly differ with respect to age, sex, growth type, histopathology, or submucosal fibrosis (Table 1). Approximately half of ESDs were performed by trainees: 13 (44.8%) in the Gi‐ESD group and 118 (51.1%) in the c‐ESD group.

**TABLE 1:** Clinicopathological characteristics of the patients and lesions.

Patient characteristics			
No. of patients	223		
Sex, male, *n* (%)	129 (57.8)		
Age, median [IQR], years	71 [62.5, 76]		

**Lesion characteristics**	**Gi‐ESD**	**c‐ESD**	***p*‐Value**
No. of lesions	29	231	
Location of the lesions, *n* (%)			0.013
Cecum and ascending colon	16 (55.2)	72 (31.2)	
Transverse colon to rectum	13 (44.8)	159 (68.8)	
Localization details			0.086
Rectum	2 (6.9)	46 (19.9)	
Sigmoid colon	6 (20.7)	52 (22.5)	
Descending colon	1 (3.4)	19 (8.2)	
Transverse colon	4 (13.8)	42 (18.2)	
Ascending colon	7 (24.1)	45 (19.5)	
Cecum	9 (31.0)	27 (11.7)	
Circumferential location, *n* (%)			
Opposite side of gravity	0	55 (23.8)	0.001
Growth type			0.21
LST‐G	10 (34.5)	54 (23.4)	
LST‐NG	15 (51.7)	105 (45.5)	
Polypoid	3 (10.3)	60 (26.0)	
Others	1 (3.4)	12 (5.2)	
Histopathology			0.74
Adenoma	18 (62.1)	115 (49.8)	
Tis	4 (13.8)	39 (16.9)	
T1a	1 (3.4)	4 (1.7)	
T1b	1 (3.4)	22 (9.5)	
SSL	4 (13.8)	39 (16.9)	
NET	1 (3.4)	12 (5.2)	
Tumor size, median [IQR], mm	25 [20, 30]	18 [13, 25]	0.001
Major diameter of resected specimen, median [IQR], mm	35 [27, 42]	27 [22, 33.5]	0.001
Minor diameter of resected specimen, median [IQR], mm	30 [23, 35]	22 [18. 27]	<0.001
Submucosal fibrosis			1
None	10 (34.5)	84 (36.4)	
Moderate‐severe	19 (65.5)	147 (63.6)	
Difficult case	7 (24.1)	22 (9.5)	0.028
Reason for ESD difficulty			0.32
Lesions extending to the appendiceal orifice	3 (10.3)	4 (1.7)	
Circumferential range ≥2/3	1 (3.4)	5 (2.2)	
Lesions extending to the ileocecal valve	2 (6.9)	1 (0.4)	
Lesions extending to a diverticulum	1 (3.4)	1 (0.4)	
Lesions extending to the dentate line	0 (0)	5 (2.2)	
Lesions extending to a scar	0 (0)	3 (1.3)	
MRS positive	0 (0)	1 (0.4)	
Lesions extending to tattooing	0 (0)	2 (0.9)	
Combined use of snare	0 (0)	5 (2.2)	1

Values are presented as median (interquartile range) or *n* (%).

Abbreviations: c‐ESD, conventional endoscopic submucosal dissection; ESD, endoscopic submucosal dissection; Gi‐ESD, gel‐immersion endoscopic submucosal dissection; LST‐G, laterally spreading granular tumor; LST‐NG, laterally spreading nongranular tumor; MRS, muscle‐retracting sign; NET, neuroendocrine tumor; SSL, sessile serrated lesion.

### 3.2 Procedural Outcomes of Gi‐ESD and c‐ESD

The median procedure time under the gel was 41 min, which was 87% of the total procedure time. No significant differences in the incidence of intraoperative perforation, delayed perforation, or postoperative hemorrhage were observed (Table 2). One patient with delayed bleeding in the Gi‐ESD group consumed alcohol on the day of discharge, which resulted in bleeding from the post‐ESD ulcer.

**TABLE 2:** Outcomes of endoscopic submucosal dissection (ESD).

Outcomes of ESD	Gi‐ESD	c‐ESD	*p*‐Value
No. of lesions	29	231	
Procedure time, min	50 [41, 77]	32 [20.5, 50.5]	<0.001
Procedure time under the gel, min	41 [22, 60]	0	
Percent procedure time under the gel (%)	89.5 [44.4, 100]	0	
Dissection speed, mm^2^/min	14 [11.4, 19.6]	16.1 [10.75, 24.4]	0.45
En bloc resection	29 (100)	229 (99.1)	1
Histologic R0 resection	28 (96.6)	205 (88.7)	0.33
Adverse events			
Intraoperative perforation	0 (0)	1 (0.4)	1
Delayed perforation	0 (0)	0 (0)	1
Delayed bleeding	1 (3.4)	1 (0.4)	0.21
Gel‐related adverse events	0 (0)	0 (0)	1

Values are presented as median (interquartile range) or *n* (%).

Abbreviations: c‐ESD, conventional endoscopic submucosal dissection; Gi‐ESD, gel‐immersion endoscopic submucosal dissection.

The procedure time was significantly longer in the Gi‐ESD group; however, there was no significant difference in dissection speed. The en bloc or histologic R0 resection rates also showed no significant differences. The histologic R0 resection rate obtained in this study was 96.6% in the Gi‐ESD group (*n* = 29) and 88.7% in the c‐ESD group (*n* = 231). Post‐hoc power analysis based on the sample size and observed effect size estimated a power of 0.06 for a two‐sided test (α = 0.05). In one case in the Gi‐ESD group, the large specimen resected en bloc became divided into two sections during retrieval through a stenosed anal canal, resulting in an RX resection.

### 3.3 Reasons for Gel Application

Among the 29 cases, gel was used to improve the visual field and buoyancy traction on the gravity side of the lesion in 14 cases(48.3%); to secure the visual field in cases where the lesion could not be approached without degassing, such as behind the folds, hepatic flexure, or splenic flexure in nine cases (31%); and allow the scope to be parallel to the muscularis propria by degassing for lesions that face the endoscope perpendicularly under CO_2_ insufflation, such as cecum lesions in six cases(20.7%). Gel was used in the entire procedure for only four of the 14 lesions on the gravity side. In the other 10, buoyancy traction was used under the gel for submerged areas, and gravity traction was used under CO_2_ for non‐submerged areas. Of the nine lesions that could not be approached without degassing, a gel was used for the entire procedure in four cases. In the other five cases, treatment was started under CO_2_, and gel was used as a rescue strategy when the lesion became difficult to approach behind the folds or flexure. For the six lesions that faced the endoscope perpendicularly, a gel was used for the entire procedure (Table 3).

**TABLE 3:** Reasons for gel application and partial gel use.

Reasons for gel application	29
1. To improve the visual field and buoyancy traction for submerged areas	14 (48.3)
Partial gel use for partially submerged lesions	10
Entire lesion submerged	4
2. To approach lesions in areas that are difficult to approach without degassing	9 (31)
Planned gel use for the entire procedure	4
Partial gel use for rescue in case of approach improvement	5
3. To approach in parallel for lesions that face the endoscope perpendicularly under CO_2_	6 (20.7)
Planned gel use for the entire procedure	6
Partial gel use for rescue in case of approach improvement	0

Values are presented as *n* (%).

### 3.4 Sensitivity Analysis

Based on propensity score matching, 23 cases in the Gi‐ESD group and 46 in the c‐ESD group were matched. The baseline characteristics of the matched cohort are shown in Supplemental Table 1. In the propensity score‐matched cohort, the histologic R0 resection rate was significantly higher in the Gi‐ESD group than in the c‐ESD group. The procedure time was significantly longer in the Gi‐ESD group than in the c‐ESD group; however, there was no significant difference in dissection speed between the two groups (Table 4).

**TABLE 4:** Outcome of the propensity score‐matched cohort.

	Gi‐ESD	c‐ESD	*p*‐Value
No. of lesions	23	46	
Procedure time, min	45 [40.5, 70]	29.5 [17.3, 54]	0.006
Dissection speed, mm^2^/min	14.9 [10.4, 19.7]	19.2 [12.1, 26.8]	0.19
En bloc resection	23 (100)	46 (100)	1
Histologic R0 resection	23 (100)	38 (82.6)	0.045
Adverse events			
Intraoperative perforation	0 (0)	1 (2.2)	1
Delayed perforation	0 (0)	0 (0)	1
Delayed bleeding	1 (4.3)	0 (0)	0.33
Gel‐related adverse events	0 (0)	0 (0)	1

Values are presented as median (interquartile range) or *n* (%).

Abbreviations: c‐ESD, conventional endoscopic submucosal dissection; Gi‐ESD, gel‐immersion endoscopic submucosal dissection.

In the c‐ESD group, the histologic R0 resection rate was significantly lower in the difficult cases, such as lesions located on the oral side of the ascending colon, lesions located on the gravity side, and large tumor size, whereas; in the Gi‐ESD group, the histologic R0 resection rate did not differ significantly across the lesion and procedural characteristics (Table 5).

**TABLE 5:** Histologic R0 resection rates stratified by lesion and procedural characteristics in the gel‐immersion endoscopic submucosal dissection (Gi‐ESD) and conventional endoscopic submucosal dissection (c‐ESD) groups.

	Gi‐ESD			c‐ESD		
	R0	R1/RX		R0	R1/RX	
	*n* = 28	*n* = 1	*p*‐Value	*n* = 205	*n* = 26	*p*‐Value
Difficult case						
Without ESD difficulty	22 (100)	0 (0)	0.24	190 (90.9)	19 (9.1)	0.005
Difficult case	6 (85.7)	1 (14.3)		15 (68.2)	7 (46.7)	
Submucosal fibrosis						
None	9 (90)	1 (10)	0.35	77 (91.7)	7 (8.3)	0.39
Moderate‐severe	19 (100)	0 (0)		128 (87.1)	19 (12.9)	
Location of the lesions						
Cecum and ascending colon	16 (100)	0 (0)	0.45	59 (81.9)	13 (18.1)	0.04
Transverse colon to rectum	12 (92.3)	1 (7.7)		146 (91.8)	13 (8.2)	
Operator						
Only trainer	15 (93.8)	1 (6.3)	1	100 (88.5)	13 (11.5)	1
With trainee	13 (100)	0 (0)		105 (89)	13 (11.1)	
Tumor size						
<20mm	7 (100)	0 (0)	1	120 (94.5)	7 (5.5)	0.003
≧20mm	21 (95.5)	1 (4.5)		85 (81.7)	19 (18.3)	
Circumferential location						
Gravity side	28 (96.6)	1 (3.4)	1	152 (85.4)	24 (13.6)	0.049
Opposite side of gravity	0(0)	0(0)		53 (96.4)	2 (3.6)	

Values are presented as *n* (%).

Abbreviations: c‐ESD, conventional endoscopic submucosal dissection; Gi‐ESD, gel‐immersion endoscopic submucosal dissection.

## 4 Discussion

In this study, Gi‐ESD was performed more frequently for lesions in submerged areas of the oral colon, such as the cecum and ascending colon. The Gi‐ESD group had a significantly larger size and a significantly greater proportion of patients with factors associated with difficult ESD than the c‐ESD group. The Gi‐ESD group had a significantly longer procedure time; however, no significant difference in dissection speed was found. Both Gi‐ESD and c‐ESD had low incidence rates of perforation and postoperative hemorrhage, and there were no adverse events specific to gel use. Propensity score matching revealed a significantly higher histologic R0 resection rate in the Gi‐ESD group, which may be attributable to the enhanced visibility provided by the gel. In the c‐ESD group, the histologic R0 resection rate was significantly lower in difficult cases, located at the cecum and ascending colon, locating gravity‐side, and large tumor size.

While some case reports have described colorectal Gi‐ESD [4, 6], there have been no reports of clinical studies comparing Gi‐ESD with colorectal c‐ESD. Maruyama et al. reported a case in which gel immersion improved the visibility during ESD for an ascending colonic lesion with continuous bleeding. In a case report of Gi‐ESD for an ascending colon lesion by Nakano et al., in addition to improved visualization during bleeding, buoyancy of the gel‐facilitated approach to the submucosa was observed. For esophageal ESD, a single‐arm study of 13 cases of esophageal Gi‐ESD for submerged left‐sided lesions reported that R0 resection was performed in all cases with no complications [19]. This study reported that Gi‐ESD resulted in less patient distress due to the avoidance of excessive air delivery, in addition to buoyancy generation and a good visual field.

In our study of colorectal ESD, while the Gi‐ESD group was performed for lesions associated with factors that make ESD difficult, a high rate of R0 resection was achieved, without an increase in complications. In colorectal Gi‐ESD, the lower endoluminal pressure with the use of a gel enables straightening and shortening of the intestinal tract for an easy approach to the lesion. In addition, by lowering the endoluminal pressure during Gi‐ESD, the lesion could be approached in the parallel direction to the muscularis propria, even for lesions that could only be viewed perpendicularly under gas insufflation.

Colonic ESD is time‐consuming, and patient movement increases the risk of perforation, particularly given the thinness of the colonic wall. Therefore, in this study, ESD was performed under sedation, using the position that provided the best scope maneuverability. Positional changes are effective in moving the lesion to the gravitationally dependent side, and from the perspective of gravity alone, the use of GEL may not be necessary. The utility of GEL in colonic ESD performed without sedation‐where patient positioning can be freely adjusted‐remains a subject for future investigation.

Traction devices have been attempted to overcome difficult lesions during colorectal ESD. Various traction devices have been developed [20, 21, 22]; however, the setup can be time‐consuming, and the traction device may interfere with the endoscope [23].

The gel can be applied with a syringe through the endoscopic channel, and it is easier than performing posture changes and setting up a traction device. The gel creates anti‐gravity traction on the submerged lesion, eliminating the need for postural changes. Removal of the gel by suction also allows the procedure to revert to gravity traction. In difficult ESD lesions, gel use may be appropriate prior to posture change or traction device use. Gel can be used as a rescue strategy during c‐ESD if it has become difficult.

This study has several limitations. First, this was a single‐center retrospective study. Therefore, the number of cases was insufficient and was not operator‐limited. The post‐hoc estimated power of 0.06 suggests that the sample size of this study may have been insufficient to detect a significant difference. Second, there was a selection bias because Gi‐ESD was performed on lesions that favor the use of gels, which are also a difficult factor for ESD. Third, since our hospital is not proficient in endoscopic mucosal resection (EMR) for large lesions, lesions suspected of cancer were more likely to be treated with ESD rather than EMR, even if they were relatively small in size. As a result, the c‐ESD group included many lesions with a small tumor size.

In conclusion, the use of gel during ESD (1) improved the visual field and buoyancy traction of submerged lesions, (2) enabled access to lesions that could not be approached with gas insufflation, and (3) allowed the scope to approach the lesion in a parallel direction to the muscularis propria, even for lesions that faced perpendicularly when under insufflation. Gi‐ESD demonstrated a high histologic R0 resection rate for large lesions with difficult features without a reduction in dissection speed.

## Author Contributions

**Kenji Yamauchi**: conceptualization (lead); investigation (lead); formal analysis (lead); writing—original draft preparation (lead), **Tomoki Inaba**: supervision(lead); writing—original draft preparation (equal), **Takeshi Morimoto**: formal analysis (equal); writing—original draft preparation (equal), **Hugh Shunsuke Colvin**: writing—original draft preparation (equal), **Akira Nakanishi**: writing—review & editing (equal), **Shigenao Ishikawa**: writing—review & editing (equal), **Masaki Wato**: writing—review & editing (equal). All authors have read and approved the final version of this manuscript.

## Ethics Statement

**Approval of the research protocol by an Institutional Review Board**: All procedures followed were in accordance with the ethical standards of the responsible committee on human experimentation (institutional and national) and the Helsinki Declaration of 1964 and later versions. The Institutional Review Board of the Kagawa Prefectural Central Hospital approved the study protocol (approval number 1127).

## Consent

Written informed consent was substituted by the opt‐out method because we used clinical information obtained in routine clinical practice, and no patients refused to participate in the study during the opt‐out period.

## Conflicts of Interest

The authors declare no conflicts of interest.

## Clinical Trial Registration

N/A.

## Supporting information




**Table S1** Clinicopathological characteristics of the matched lesions.

**Video S1** Gel‐immersion endoscopic submucosal dissection for a laterally spreading tumor (granular type, 30 mm in diameter) located on the ascending colon.
