# Supplementary material for: Gel‐immersion Endoscopic Submucosal Dissection for Superficial Colorectal Neoplasms: A Retrospective Study Comparing Conventional Endoscopic Submucosal Dissection
Source: DEN Open. 2025 Oct 10;6(1):e70221. doi: 10.1002/deo2.70221 (PMC12511952; doi:10.1002/deo2.70221)
Supplement: Supplementary file 1 — Table S1 Clinicopathological characteristics of the matched lesions. [file DEO2-6-e70221-s001.docx]

**Supplemental Table 1** Clinicopathological characteristics of the matched lesions

| Lesion characteristics | | Gi-ESD | c-ESD | *P* value |
| --- | --- | --- | --- | --- |
| No. of lesions | | 23 | 46 |  |
| Location of the lesions, *n* (%) | |  |  | 1 |
|  | Oral side from the ascending colon | 11(48.7) | 23(50) |  |
|  | Anorectal side from the transverse colon | 12(52.2) | 23(50) |  |
| Localization details | |  |  | 1 |
|  | Rectum | 2 (8.7) | 5 (10.9) |  |
|  | Sigmoid colon | 6 (26.1) | 10 (21.7) |  |
|  | Descending colon | 1 (4.3) | 1 (2.2) |  |
|  | Transverse colon | 3 (13.0) | 7 (15.2) |  |
|  | Ascending colon | 7 (30.4) | 13 (28.3) |  |
|  | Cecum | 4 (17.4) | 10 (21.7) |  |
| Circumferential location, *n* (%) | |  |  |  |
|  | Opposite side of gravity | 0 | 0 | 1 |
| Growth type | |  |  | 0.35 |
|  | LST-G | 8 (34.8) | 514 (30.4) |  |
|  | LST-NG | 12 (52.2) | 19 (41.3) |  |
|  | Polypoid | 2 (8.7) | 12 (26.1) |  |
|  | Others | 1 (3.4) | 1 (2.2) |  |
| Histopathology | |  |  | 0.74 |
|  | Adenoma | 14 (60.9) | 20 (43.5) |  |
|  | Tis | 3 (13.0) | 8 (17.4) |  |
|  | T1a | 1 (4.3) | 1 (2.2) |  |
|  | T1b | 1 (4.3) | 2 (4.3) |  |
|  | SSL | 3 (13.0) | 14 (30.4) |  |
|  | NET | 1 (3.4) | 1 (2.2) |  |
| Tumor size, median [IQR], mm | | 23 [17.5, 29] | 20 [16, 25.8] | 0.32 |
| Major diameter of the resected specimen, median [IQR], mm | | 34 [26, 42] | 27.5 [23, 35] | 0.08 |
| Minor diameter of the resected specimen, median [IQR], mm | | 27 [22.5, 35] | 22.5 [17.5, 26.8] | 0.02 |
| Submucosal fibrosis | |  |  | 0.8 |
|  | None | 8 (34.8) | 18 (39.1) |  |
|  | Moderate-severe | 15 (65.2) | 28 (60.9) |  |
| Difficult case | | 2 (8.7) | 3 (6.5) | 1 |
| Reason for ESD difficulty | |  |  | 1 |
|  | Circumferential range ≥2/3 | 0 (0) | 1 (33.3) |  |
|  | Lesions extending to the ileocecal valve | 1 (50) | 1 (33.3) |  |
|  | MRS positive | 0 (0) | 1 (33.3) |  |
|  | Lesions extending to the appendiceal orifice | 1 (50) | 2 (0.9) |  |

Values are presented as median (interquartile range) or *n* (%).

ESD, endoscopic submucosal dissection; Gi-ESD, gel-immersion endoscopic submucosal dissection; c-ESD, conventional endoscopic submucosal dissection; LST-G, laterally spreading granular tumor; LST-NG, laterally spreading nongranular tumor; SSL, sessile serrated lesion; NET, neuroendocrine tumor; MRS, muscle-retracting sign;
